# Evaluating the Difference between Virtual and Paper-Based Clinical Cases in Family Medicine Undergraduate Education

**DOI:** 10.1155/2018/1408450

**Published:** 2018-01-15

**Authors:** Zalika Klemenc-Ketis, Branka Cagran, Dejan Dinevski

**Affiliations:** ^1^Faculty of Medicine, University of Maribor, Taborska 8, Sl-2000 Maribor, Slovenia; ^2^Department of Family Medicine, Faculty of Medicine, University of Ljubljana, Poljanski nasip 58, Sl-1000 Ljubljana, Slovenia; ^3^Community Health Centre Ljubljana, Metelkova 9, Sl-1000 Ljubljana, Slovenia; ^4^Faculty of Education, University of Maribor, Koroska cesta 160, Sl-2000 Maribor, Slovenia

## Abstract

**Introduction:**

A “virtual patient” is defined as a computer program which simulates real patients' cases. The aim of this study was to determine whether the inclusion of virtual patients affects the level of factual knowledge of family medicine students at the undergraduate level.

**Methods:**

This was a case-controlled prospective study. The students were randomly divided into experimental (EG: *N* = 51) and control (CG: *N* = 48) groups. The students in the EG were asked to practice diagnosis using virtual patients instead of the paper-based clinical cases which were solved by the students in the CG. The main observed variable in the study was knowledge of family medicine, determined by 50 multiple choice questions (MCQs) about knowledge of family medicine.

**Results:**

There were no statistically significant differences in the groups' initial knowledge. At the final assessment of knowledge, there were no statistically significant differences between the groups, but there was a statistically significant difference between their initial and final knowledge.

**Conclusions:**

The study showed that adding virtual patient cases to the curriculum, instead of paper clinical cases, did not affect the level of factual knowledge about family medicine. Virtual patients can be used, but a significant educational outcome is not expected.

## 1. Introduction

Computer-assisted learning (CAL) is defined as any use of computers, multimedia, or interactive technologies for aiding or supporting education and training [1]. Studies have shown that CAL is significantly more successful than traditional methods of teaching in terms of better student knowledge, performance, and satisfaction [1, 2]. CAL is not an independent teaching method, but rather a combination of different forms of instruction, and therefore must be integrated within the system of education [3–5].

“Virtual patients” are one of the methods of CAL. They are built on the theory of computer games to be used in medical education. The educational concept of virtual patients is based on the theory of constructivism [6], which emphasises the meaning of experience and self-assessment. Experience, even though it is indirect, and prompt self-assessment are strong features of virtual patients. A virtual patient is defined as a computer program which simulates real patients' cases and hence enables the students to gather case histories, perform clinical examinations, and decide on diagnostics and therapy [7]. The simulation of clinical cases on a computer screen has been available since the 1970s [8], but virtual patients, as defined by Cook and Triola, were implemented within medical education much later.

The most important feature of virtual patients is a clinical scenario which enables an interactive computer simulation of patients focused on teaching and assessing the students [9]. The use of virtual patients offers the students a realistic learning environment which is repeatable and can have different difficulty levels [9]. Virtual patients can also be used for assessment, both formative and summative. Formative assessment can be made with continuous feedback to students about their performance. There are several tools integrated within the cases for this purpose: multiple choice questions, summary statement feedback, a case analysis tool, and so on. For summative assessment, comprehensive multiple choice exams are available [10].

There are several ways of integrating virtual patients into the existing medical education curriculum at the undergraduate level. They can be used as a form of self-directed learning, group learning, or a combination of face-to-face learning and e-learning, among others [9]. Virtual patients can be used as a preparation tool for clinical clerkships, as well as learning about patient treatments not usually encountered in everyday practice. Regardless of the means of implementation, students benefit from acquiring skills through multifaceted virtual patients [11].

The use of virtual patients in primary care and family medicine has already been described [12]. A previous study on the attitudes of students towards the use of virtual patients during undergraduate courses in family medicine showed that students accepted this teaching method with great interest and saw a potential usefulness in their studies [13]. The work of a family physician is based on six main competencies that are defined in the European definition of general practice/family medicine [14]: primary care management, person-centred care, specific problem-solving skills, a comprehensive approach, community orientation, and a holistic approach. It is therefore essential that family medicine education follows these special guidelines [15] when virtual patients are used. This has already been shown by some authors, who have stated that teaching family medicine through virtual patients is of a different nature than that of other clinical specialities [12, 16]. Specifically, students need to learn not only about the clinical management of the patients but also about communication, patient-centred thinking, and the comprehensive and holistic management of the patients [12, 16].

As there are only a few studies dealing with the use of virtual patients in family medicine undergraduate education, and there is a lack of evidence on how it affects students' knowledge of family medicine, we decided to carry out some experimental research. We wanted to determine whether the inclusion of virtual patients affects the level of factual knowledge of family medicine at the undergraduate level.

## 2. Materials and Methods

### 2.1. Type of Study and Settings

This was a case-controlled prospective study which took place at the Medical Faculty of the University of Maribor in Slovenia in the study year 2014/2015, during the course in family medicine at the undergraduate level.

### 2.2. Participants

The research population consisted of all undergraduate students who had enrolled in the 4th study year in 2014/2015 who attended the obligatory course on family medicine I (*N* = 99). We randomly assigned the students to two groups: experimental (EG) (*N* = 50) and control (*N* = 49) (CG) group.

### 2.3. Experiment

The teaching of family medicine in the 4th study year (7th semester, four months) consists of lectures, seminars, and different exercises [17]. In one of the exercises, the students receive paper-based clinical cases for which they must produce a written assignment on how they plan to manage this patient. In this study, the students in the EG had to solve virtual patient cases instead of the paper-based clinical ones.

Virtual patients are available through the MedU platform [10]. The clinical cases on this platform are developed for different medical specialties, including family medicine. The cases are built on the collaborative development model [18].

The students in the EG had to solve five virtual patient cases chosen by the lecturer in order to be in line with the learning objectives of the subject. Each student had to solve the same five virtual patient cases. The students in the CG had to solve five paper-based clinical cases, which were the same as the virtual ones but presented in written form. The cases were a 65-year-old woman with insomnia, a 24-year-old woman with headache, a 42-year-old man with pain in the right upper abdomen, a 55-year-old man with fatigue, and a 30-year-old woman with palpitations.

The virtual patient case comprised 15–30 pages in which students assume the role of a virtual student working with a preceptor. The students move through the stages of a patient's presentation, engaged in continuous practice of their clinical reasoning skills, from eliciting the main complaint to taking a history, performing a physical exam, composing an assessment, formulating a differential diagnosis, and on to diagnostic testing and management. The methods applied consist of reading additional material (articles, textbook chapters), photographic images of patients during a physical exam, video clips of topics associated with the case, and so on.

The paper-based clinical case consisted of patient history, results from a clinical examination, and investigations. No additional material was provided.

### 2.4. Data Collection

Data was collected by paper forms consisting of demographic data (gender and age of the students), a learning strategies scale, a learning habits scale, and a knowledge of family medicine scale. We collected data in two phases: (1) before the beginning of the experiment (in October 2014) we obtained data on the students' initial knowledge of family medicine, learning strategies, and learning habits and (2) at the end of the experiment, after four months of teaching (in February 2015), we checked the students' final knowledge of family medicine.

#### 2.4.1. Test of the Initial and Final Knowledge of Family Medicine

Both tests included 50 multiple choice questions (MCQs) on factual knowledge of family medicine, and the same MCQs were used for the initial and the final knowledge tests. The maximum number of points in each test was 50. The MCQ questions were based on the learning outcomes of the family medicine subject in the 4th study year and were taken from the textbook of family medicine used in the curriculum as the obligatory learning source [19].

#### 2.4.2. Scale for Assessing Learning Strategies

This scale is composed of 20 statements covering four sets of learning strategies: strategies of conditions management (e.g., real-time learning, time distribution); strategies of processing learning material (e.g., repetition of old material, independent renewal, and linking courses); metacognitive strategies (e.g., self-questioning); and strategies of controlling emotional motivation states (e.g., concentration in learning). The statements can be rated on a 3-point scale: 1—disagree, 2—partly agree, and 3—agree. The scale's total score is 60 points; a higher total result indicates a better mastery of the internal and external factors of learning and a better processing of learning material and hence a higher quality (durability, usability) of knowledge.

#### 2.4.3. Scale for Assessing Learning Motivation

This scale is composed of 20 statements which assess internal learning motivation positively and negatively (e.g., self-initiative learning; learning beyond others' demands; striving for knowledge rather than marks). The statements can be rated on a 3-point scale: 1—disagree, 2—partly agree, and 3—agree. The scale's total score is 60 points; a higher total outcome in the scale denotes a higher level of internal learning motivation.

Both scales are established tools for the measurement of learning motivation and strategies and have been proven to be valid and reliable [20, 21]. Both scales have been adapted for the Slovenian language and have proved to be reliable [22].

### 2.5. Statistical Analysis

Data was processed at the level of descriptive and inferential statistics. The following methods were applied: descriptive statistics for sample description; independent samples *t*-test and paired samples *t*-test for analyses of differences between groups; analysis of covariance (ANCOVA) for homogeneity; and effect size measures (*η*^2^, *R*^2^) for determining the size of the study effect. The reliability of the scales was assessed by Cronbach's alpha. A *P* value of <0.05 was considered as statistically significant.

## 3. Results

### 3.1. Demographic Characteristics

There were 68 (68.7%) female students in the sample. In the experimental group, there were 39 (76.5%) women and in the control group 29 (60.4%) women. The mean age of the participants was 22.2 ± 1.0 years. There were no statistically significant differences between the experimental and control groups in terms of gender and age.

### 3.2. Reliability of the Instruments

The Cronbach's alpha of the initial knowledge test was 0.843, of the learning strategies scale 0.823, and of the learning motivation scale 0.793.

### 3.3. Initial Assessment of Learning Strategies, Learning Motivation, and Knowledge of Family Medicine

The students demonstrated an average level of functioning from the viewpoint of all three factors of the initial status (knowledge, learning strategies, and learning motivation): the score for knowledge was 22.0 ± 3.5, for learning 42.2 ± 4.4, and for learning motivation 46.4 ± 4.5.

There was no statistically significant difference between the EG and the CG in their initial knowledge of family medicine, in their level of learning strategies, or in the level of their internal learning motivation (Table 1).

### 3.4. Final Assessment of Learning Strategies, Learning Motivation, and Knowledge of Family Medicine

In the final assessment of knowledge of family medicine, the students achieved a total of 31 to 49 points. The mean score was 40.9 ± 4.2 points, the skewness was −0.5, and the kurtosis was 0.4.

The *F* test of differences between the means showed no statistically significant differences between the EG and the CG in terms of family medicine knowledge, learning strategies, or learning motivation (Table 2).

There was a statistically significant difference between the initial and final knowledge of family medicine. The EG and CG students were almost equal in the level of their attained knowledge of family medicine (Table 3).

## 4. Discussion

To the best of our knowledge, this was the first study assessing the effects of the use of virtual patients in the undergraduate teaching of family medicine at the level of factual knowledge in family medicine. It showed that adding virtual patient cases to the curriculum instead of paper clinical cases did not affect the level of factual knowledge of family medicine.

Previous studies have demonstrated that the main usefulness of virtual patients can be seen as a form of effective learning of clinical reasoning [7, 23–25]. Our study did not evaluate this, but instead it evaluated factual knowledge. A randomised controlled trial by Vash et al. [26] showed that the students who used virtual patients during their undergraduate surgery education significantly improved in history taking, but no differences were found regarding the appropriate use of tests or in proposing differential diagnoses and management plans. In addition, Stevens et al. [27] reported that virtual clinical scenarios in surgery teaching could provide students with a controllable, secure, and safe learning environment, but this study was not experimental. Overall, the use of virtual patients in surgery teaching is effective but due to the very small number of randomised controlled trials on the topic more evidence is needed [3]. Similarly, the use of virtual patients in pharmacy education seems to be successful, particularly for optimising the teaching process, but in order to draw accurate conclusions further appropriate studies should be made [28].

Based in the results of our study, we can say that the students who solved virtual patient cases did not achieve significantly higher levels of knowledge of family medicine when compared to the students who solved paper clinical cases. Here, it should be mentioned that the students' level of knowledge significantly improved after the end of the family medicine course, regardless of whether they were in the EG or the CG. This shows that the curriculum with virtual patients and the curriculum with paper clinical cases are equal in terms of acquiring knowledge of family medicine during the undergraduate course and can be included in the curriculum. If the results of our study had shown that students who solved clinical cases through virtual patients achieved significantly higher levels of factual knowledge than other students, this would have indicated the need to replace paper clinical cases with virtual clinical cases in order to achieve better academic performance.

When evaluating the use of virtual patients in education, we should take into account the results of previous studies, which showed that the sound effects and animation used in e-materials distract attention, reduce working memory and consequently lower the level of reading comprehension, while increasing stress [29]. It should be noted that in our case the fact-based data acquired by students was used as the effect indicator for learning family medicine with virtual patients. The results of our study showed that using e-materials had no specific advantages or disadvantages when compared to paper-based material. However, advantages over the traditional (printed) material could show up at other levels, such as clinical reasoning or clinical competences [30]. For this reason, it would make sense to check the efficiency of the use of virtual patients at other levels and from other viewpoints and use different tools.

We included the assessment of learning strategies and motivation in the study as it has already been shown that they are positively related to academic performance [31, 32]. The results showed that the EG and the CG did not differ significantly in terms of learning strategies and motivation. This is important as the possible influence of different levels of these variables could have had a confounding effect.

The strength of this study was its methodology, which enabled us to study causal relationships, the homogeneity of our sample, and the fact that all the enrolled students took part in the study. The fact that our study sample was homogenous and there were no significant differences between the CG and the EG in terms of basic features (gender, knowledge, learning strategies, and learning motivation) provided an important positive starting point for checking the effects of the experiment, that is, the effects of the use of virtual patients in the family medicine course on students' factual knowledge. Also, all the tools used proved to be reliable as their Cronbach's alpha was above the recommended value of 0.7 [33]. This gives additional value to the results.

It may, however, have happened that differences between the CG and the EG went undetected due to an insufficient sample size, an insufficient number of virtual patients which the students had to solve, a lack of enthusiasm in the EG students, or a lack of sensitivity in the MCQ test. Also, using the same MCQs before and after test may have a substantial effect on the outcome and could minimize the difference in the effect of the VP versus paper groups. These facts could be the limitations of our study, which should be taken into account when interpreting the results.

## 5. Conclusions

Based on the results of our study, we can confirm that virtual patients can be used during the teaching of family medicine at the undergraduate level, but our results did not show any added value of their use. As there are only a few studies in the literature dealing with the use of virtual patients in undergraduate medical education, our study adds important knowledge to this topic. It also confirms the need for large randomised controlled trials which will enable us to draw firmer conclusions.

## Figures and Tables

**Table 1:** Results of the *t*-test for independent samples of differences in the total score of the initial tests of knowledge, learning strategies, and learning motivation per groups.

Factors of the initial status	Group	Mean	Standard deviation	Test of variances' homogeneity		Test of means' differences	
				*F*	*P*	*t*	*P*
Knowledge	Control	22.2	3.7	1.241	0.268	0.655	0.514
	Experimental	21.8	3.4				
Learning strategies	Control	41.5	4.6	1.059	0.306	1.562	0.122
	Experimental	42.9	4.1				
Learning motivation	Control	46.1	4.7	1.285	0.260	0.750	0.455
	Experimental	46.8	4.3				

**Table 2:** Results of the ANCOVA of the differences between the EG and the CG in the total score of the family medicine knowledge test after the experiment, with the criterion variable in controlling the previous knowledge in family medicine as a covariable.

Group	Mean	Standard deviation	Test of homogenity of variances		Test of the homogenity of regression coefficient		Test of differences between means		Effect size measure
			*F*	*P*	*F*	*P*	*F*	*P*	*η* ^2^
Control Experimental	40.9	4.4	0.269	0.605	0.424	0.517	0.076	0.783	0.004
	40.9	4.1							

**Table 3:** Results of the paired samples *t*-test of differences in the total result in the EG and the CG students' FM knowledge test before and after the experiment.

Group	Factor	Mean	Standard deviation	Means' difference	Test of differences between means	
					*t*	*P*
Control	Initial knowledge	22.2	3.7	18.7	28.157	<0.001
	Final knowledge	40.9	4.4			
Experimental	Initial knowledge	21.8	3.4	19.2	35.027	<0.001
	Final knowledge	40.9	4.1			

## Abbreviations

EG: Experimental group
CG: Control group
CAL: Computer-assisted learning
MCQ: Multiple choice question
ANCOVA: Analysis of covariance.
